# Osteocytes: master orchestrators of skeletal homeostasis, remodeling, and osteoporosis pathogenesis

**DOI:** 10.3389/fcell.2025.1670716

**Published:** 2025-09-25

**Authors:** Yan Wu, Donghao Gan, Zhikang Liu, Daodi Qiu, Guoqing Tan, Zhanwang Xu, Haipeng Xue

**Affiliations:** ^1^ The First Affiliated Hospital of Shandong First Medical University, Jinan, China; ^2^ Yale School of Medicine, New Haven, CT, United States; ^3^ Shandong University of Traditional Chinese Medicine, Jinan, China; ^4^ The Affiliated Hospital of Shandong University of Traditional Chinese Medicine, Jinan, China

**Keywords:** osteocyte, bone, microenvironment, osteoporosis, fracture

## Abstract

The skeleton functions as an endocrine organ. Osteocytes maintenance of skeletal strength and energy balance by sensing mechanical stress and communicating with surrounding cells. They are currently considered key regulators of bone remodeling, mineral metabolism, and systemic homeostasis. Osteocytes originate from osteoblasts and are embedded in the lacunar-tubular network. They express proteins such as DMP1, sclerostin, and FGF23, and influence Wnt signaling, the RANKL/OPG axis, and phosphate metabolism. We review the latest studies in the field of osteocyte biology, focusing on their mechanotransduction through Piezo1 and integrins, regulation of osteoclastogenesis and osteogenesis, and their interactions with the bone marrow microenvironment, including immune and vascular cells. In osteoporosis, osteocyte dysfunction is manifested by apoptosis, ferroptosis, and pyroptosis. These changes, together with altered secretion, lead to uncoupled remodeling, disruption of the lacuno-canalicular network and metabolic imbalances that are intertwined with inflammation and bone marrow fat deposition. Osteocytes play an important role in fracture healing and adaptive remodeling under mechanical stimulation, promoting angiogenesis and stem cell recruitment. A growing number of emerging approaches, including stem cell therapy, CRISPR editing, and AI-driven multi-omics for precision medicine, are accelerating osteocyte-related research and the development of therapeutic strategies. These studies reveal the clinical potential of osteocyte-targeted therapies to prevent osteoporosis, improve bone strength, and enhance regeneration. By integrating molecular, cellular, and systems knowledge, we highlight osteocytes as a key therapeutic target to combat bone diseases and promote bone regeneration.

## 1 Introduction

Bone is far more than just a rigid support for the body. In reality, it is an active and adaptable tissue—functioning as an endocrine organ, a mineral ion reservoir, and a site of ongoing renewal that responds to mechanical forces, hormones, and changing metabolic needs (Szeliga et al., 2024). This constant process of turnover—where bone is built, broken down, and maintained—preserves skeletal strength and also helps regulate broader physiological systems, such as calcium and phosphate homeostasis and even aspects of energy balance (Zhou et al., 2021). When the balance between these processes is disturbed, serious disorders can result. Osteoporosis, for example, is marked by weak, fracture-prone bones and affects millions of people worldwide. In recent years, researchers have started to view the bone microenvironment as a highly interactive system, one that brings together a variety of cell types and complex signaling networks, opening up new possibilities for therapy (Zaidi et al., 2018).

There is great diversity in cells and signals in bone. Regions interact. Rather than existing as isolated compartments, regions like the endosteum, periosteum, marrow stroma, and the vascular network are in constant interaction, each contributing distinct cellular residents: hematopoietic stem cells, various mesenchymal and immune cells (including macrophages and T cells), endothelial cells, and adipocytes, all within an extracellular matrix rich in collagen and growth factors (Busch et al., 2024). In recent years, spatial transcriptomics and imaging have helped researchers pick apart these niches. For example, endosteal zones are generally linked to hematopoietic stem cell quiescence (with CXCL12+ stromal cells), while areas around blood vessels appear to foster osteoprogenitor differentiation through angiocrine factors (Dalle Carbonare et al., 2025). But osteocytes—so often underappreciated—seem to interact with nearly all of these other players, using gap junctions or secreted vesicles to influence both inflammation and stem cell fate. It is worth mentioning that, especially with age or in certain diseases, osteocytes can enter a senescent state, pumping out more pro-inflammatory cytokines and in turn tilting the immune environment and driving osteoclast activity (Chen S. et al., 2024). Emerging insights from recent studies highlight microbial influences, where gut-derived metabolites shape bone cell diversity, linking the microbiome to skeletal health (Dalle Carbonare et al., 2025). This multifaceted microenvironment not only sustains bone remodeling but also contributes to pathologies like osteoporosis, where dysregulated cell interactions exacerbate bone loss.

In this review, we synthesize recent advances in our understanding of osteocyte biology, with a particular focus on their roles in bone metabolism, osteoporosis, and skeletal remodeling. We begin by summarizing the developmental origins and molecular features of osteocytes, followed by an exploration of their central regulatory functions and signaling networks. We then highlight mechanisms of osteocyte dysfunction in osteoporosis and their contributions to pathological bone loss. Finally, we discuss the involvement of osteocytes in bone regeneration and remodeling, emerging regulatory pathways, and the translational potential of targeting osteocyte signaling in therapeutic strategies for metabolic bone diseases. We aim to provide a comprehensive perspective on osteocytes as master regulators of bone health and disease, and highlight osteocytes are key therapeutic targets that can help treat bone diseases and promote bone regeneration (Lu et al., 2025; Wu M. et al., 2024).

## 2 Osteocyte biology: origin, morphology, and molecular characteristics

Our understanding of osteocytes has changed a lot over time. Early on, these cells were largely regarded as quiet, “buried” components of bone, with little thought given to any active role they might play. This view was based on early histological studies from the 19th century, which described osteocytes as little more than passive managers of local mineral exchange. In truth, osteocytes are the most long-lived cells found in bone. They originate from osteoblasts as bones are laid down, eventually becoming encased within the mineralized matrix. At the same time, they develop an extensive network of dendritic processes, forming the lacuno-canalicular network (LCN) that reaches throughout the bone tissue (Dallas et al., 2013). Based on this network, osteocytes can sense mechanical changes, transport nutrients, and send paracrine signals. They are also sensitive to microdamage, variations in fluid flow, and hormonal signals. Research breakthroughs in the 1990s—driven by advances in genetic manipulation and imaging—demonstrated that osteocytes actively secrete regulatory molecules such as sclerostin (which inhibits Wnt signaling) and RANKL (which promotes osteoclast formation), confirming their central role in bone regulation (Bonewald, 2011; Delgado-Calle and Bellido, 2022). Single-cell omics and *in vivo* lineage tracing studies reveal the central role of osteocytes in processes ranging from periosteum remodeling to immune regulation (Tresguerres et al., 2020; Creecy et al., 2020).

Originating from osteoblasts, once situated within their lacunae, osteocytes take on a variety of responsibilities—such as sensing mechanical forces, managing mineral metabolism, and maintaining communication with other cells—all of which are vital for bone health. For a long time, osteocytes were considered passive “bystanders” in the skeleton. However, with the development of technologies such as single-cell omics and high-resolution imaging, researchers have gradually revealed the central role of osteocytes in bone biology (Palumbo and Ferretti, 2021).

### 2.1 Osteocyte developmental lineage and differentiation

Mesenchymal stem cells (MSCs) differentiate into the osteoblast lineage and drive maturation under the influence of key transcription factors such as Runx2 and Osterix (Sp7) (Zhu S. et al., 2024; Ponzetti and Rucci, 2021). During bone formation, osteoblasts secrete extracellular matrix (ECM), and some of these cells eventually become osteocytes as they undergo both morphological and functional changes. The transition from osteoblasts to osteocytes is characterized by a significant decrease in anabolic activity, the growth of dendritic processes, and the gradual encapsulation of the cells into pits formed in the mineralized matrix (Dallas and Bonewald, 2010; Mullen et al., 2013). This embedding is further shaped by perilacunar remodeling—a process in which osteocytes themselves take on an active role, modifying the surrounding matrix by means of osteocytic osteolysis, enabling localized bone resorption and remodeling, and also altering lacunar morphology, thereby influencing both mineral balance and the fine structure of bone (Franz-Odenda et al., 2006). Notably, this transition is coupled with changes in cellular metabolism. Glycolysis is especially important in the early stages of differentiation. Mature osteocytes, however, become more versatile in the types of fuel they use, which helps them survive in the relatively low-oxygen conditions found within the bone matrix (Prideaux et al., 2025).

Osteocytes gain biomarkers as they mature. Each biomarker links to a specific function. In differentiation, dentin matrix protein 1 (DMP1) takes on a central role in maintaining phosphate balance and facilitating matrix mineralization. It is well established that mutations in DMP1 can result in hypophosphatemic rickets as well as defects in osteocyte function (Dussold et al., 2019; Li et al., 2022). As osteocytes reach maturity, they begin to express sclerostin—known to inhibit the Wnt signaling pathway and thus limit bone formation—as well as fibroblast growth factor 23 (FGF23), which acts as a hormone regulating phosphate and vitamin D metabolism (Knowles et al., 2023; Ratsma et al., 2023; Ratsma et al., 2024). The synthesis of FGF23 in osteocytes is affected by local phosphate levels and FGFR1-mediated signaling, and studies involving targeted deletions in osteocytes have highlighted the importance of this pathway in guarding against hyperphosphatemia (Courbon et al., 2023; Xiao et al., 2014). Additionally, molecules such as Phex and Mepe serve to further characterize osteocyte identity, and their co-expression in mature osteocytes has been confirmed through single cell approaches (Prideaux et al., 2016; Hanai et al., 2023). These biomarkers do more than identify cells. For example, anti-sclerostin antibodies can increase bone mass in osteoporosis models. Other key markers include E11/gp38, which serves as an early osteocyte marker and helps with dendrite formation (Prideaux et al., 2012; Zhang et al., 2006). MT1-MMP aids in canaliculi formation during osteocyte development (Holmbeck et al., 2005; Kul et al., 2012; Karsdal et al., 2004). CapG and destrin regulate the cytoskeleton and control cytoplasmic processes in dendrites (Delgado-Calle and Bellido, 2015). Osteocytes are also richer in molecules like PHEX that control phosphate homeostasis compared to osteoblasts (Donmez et al., 2022; Rowe, 2012).

Epigenetics guide osteocyte development and function. In the context of bone, changes such as histone modification and DNA methylation are widely accepted as important drivers of lineage commitment (Park-Min, 2017; Dashti et al., 2024). The transition from osteoblasts to osteocytes is largely dependent on chromatin remodeling; enzymes such as histone deacetylases (HDACs) and the methyltransferase EZH2 (which modifies H3K27) repress genes involved in proliferation while regulating the expression of genes associated with bone formation (Zhu S. et al., 2024; Husain and Jeffries, 2017; Zhang et al., 2024). Furthermore, methylation of CpG sites within the SOST promoter has been associated with reduced sclerostin production following mechanical stimulation (Delgado-Calle et al., 2012). In osteocytes, miR-29b-3p responds to mechanical strain and regulates osteoblast differentiation by controlling IGF-1 secretion (Zeng et al., 2019). The non-coding RNA miR-218 expressed in osteocytes inhibits osteoblast differentiation and regulates its function through the Wnt pathway (Hassan et al., 2012). Meanwhile, technological advances like single-cell RNA sequencing (scRNA-seq) and spatial transcriptomics have dramatically expanded our perspective on osteocyte heterogeneity (Wang et al., 2025; Agoro et al., 2023; Feng et al., 2023). ScRNA-seq has revealed previously unrecognized, transcriptionally distinct subpopulations within the broader bone cell milieu—including not only osteocytes, but also mesenchymal stem cells, osteoblasts, chondrocytes, fibroblasts, osteoclasts, and vascular cells (Chai, 2022). Spatial transcriptomics, by mapping gene expression within intact tissue, helps clarify how these cell types interact within their microenvironments (Matsushita et al., 2023; Mathavan et al., 2025). Key transcription factors like ATF4 and HIF-1α integrate signaling pathways, with senescence-associated epigenetic changes in driving age-related dysfunction (Nusrat et al., 2025). These omics methods show the diversity of osteocytes. They help in better treatments for bone diseases.

### 2.2 Osteocyte ultrastructure and network architecture

Osteocytes are characterized by their stellate shape and extend numerous cytoplasmic processes (dendrites), which together form a complex LCN (Tiede-Lewis and Dallas, 2019; Moriishi and Komori, 2022). Within the LCN, the lacunae accommodate the osteocyte cell bodies, while the canaliculi—narrow, branching channels—permeate the mineralized matrix, thus permitting the exchange of nutrients and waste as well as fluid movement required for mechanotransduction (van Tol et al., 2020; Schemenz et al., 2020). Modern imaging methods, notably synchrotron X-ray tomography, have made it possible to appreciate how the LCN varies: for example, denser canalicular networks are generally observed in cortical bone, while the trabecular regions are less interconnected. Notably, age-related changes, such as occlusion of canaliculi, are linked with increased bone fragility (Moriishi and Komori, 2022). Shear stress generated by fluid movement within these channels can activate integrins and specific ion channels like Piezo1, leading to calcium influx and subsequent activation of pathways such as Wnt/β-catenin (Qin et al., 2020). Communication between osteocytes relies heavily on gap junctions formed by connexin 43 (Cx43), which assemble into hemichannels that permit the passage of small molecules, including ATP, prostaglandins, and cyclic nucleotides (Zhang et al., 2025). When osteocytes are subjected to mechanical loading, Cx43 hemichannels facilitate the release of prostaglandin E2 (PGE2), which can modulate the activity of nearby osteoblasts. In addition to these classic structures, extracellular vesicles and tunneling nanotubes also play a part, allowing for communication over longer distances (Delgado-Calle and Bellido, 2022). Loss of LCN connectivity—whether due to age or disease—tends to impair mechanosensation and promote cellular senescence, further emphasizing the significance of this system in preserving bone strength and integrity (Tiede-Lewis and Dallas, 2019).

## 3 Osteocyte as the master regulator of bone metabolism

Osteocytes are found deep within the bone matrix, where they play a vital role in keeping the skeleton balanced. These cells constantly process a mix of signals—mechanical loads, biochemical fluctuations, and hormonal inputs—allowing them to coordinate the complex process of bone metabolism. They are sensitive to changes in their local environment and can release various factors that act on osteoblasts and osteoclasts, ensuring that bone tissue can adjust to different physiological demands as needed (Qin et al., 2020; Choi et al., 2024). If osteocyte communication goes awry, issues like abnormal phosphate levels or a breakdown in the coupling of bone formation and resorption may develop, highlighting why these cells have become promising therapeutic targets in diseases such as osteoporosis (Michigami, 2022; Han et al., 2018).

### 3.1 Mechanosensation in osteocytes

Osteocytes sense mechanical loading and convert it into biochemical signals (Qin et al., 2020; Choi et al., 2021). Fluid shear stress stimulate mechanosensitive pathways. Among these, Piezo1 acts as a cation channel that drives calcium entry when the membrane is deformed (Li et al., 2019; Wang et al., 2023; Sun et al., 2019). Piezo1 activation in osteocytes triggers a downstream cascade, including Wnt/β-catenin signaling, which enhances osteogenesis and inhibits bone resorption, as evidenced by impaired bone formation and increased osteoclast activity in conditional knockout models (Li X. et al., 2025; Nottmeier et al., 2023). Integrins mediate mechanotransduction by linking cytoskeletal tension to focal adhesions, facilitating force transmission to the nucleus (Qin et al., 2022; Qin et al., 2023). YAP/TAZ, co-activators shuttled to the nucleus under mechanical cues, amplify this process by transcribing genes for matrix remodeling, promote perilacunar resorption to adapt to loading (Qin et al., 2020).

**FIGURE 1:**
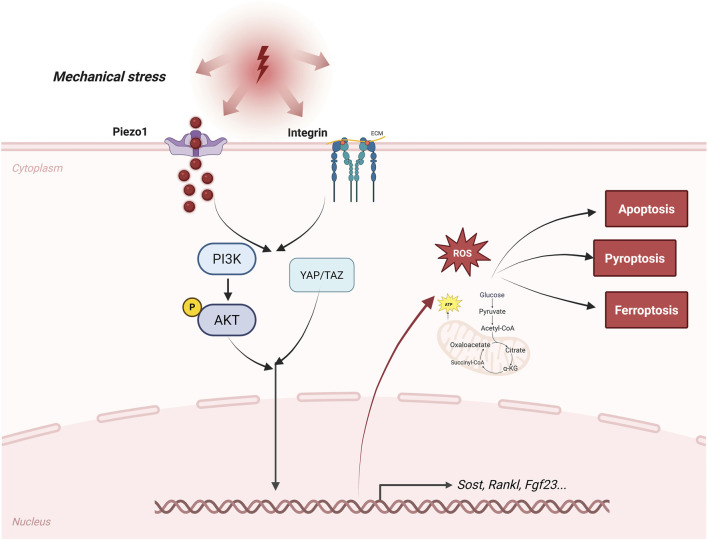
Pathway of bone cells sensing mechanical stress. This figure outlines the signaling pathways by which osteocytes sense mechanical stress. Mechanotransduction pathways, such as those involving Piezo1, integrins, YAP/TAZ, and Wnt/β-catenin, as well as paracrine and endocrine signals, such as sclerostin, RANKL, and FGF23, are depicted. The figure also labels important regulated forms of cell death, including apoptosis, ferroptosis, and pyroptosis, as well as metabolic changes, such as a shift toward glycolysis and increased production of reactive oxygen species, that contribute to osteocyte dysfunction during osteoporosis. Arrows indicate the main signaling pathways and feedback mechanisms in these processes.

The osteocyte mechanosensory network is critical for adapting bone structure to mechanical load (Figure 1). Cyclic loading of the skeleton triggers changes in osteocyte function, including cortical bone thickening, whereas cyclical reduction in bone use often leads to bone loss (Iandolo et al., 2021; Ma et al., 2023). In microgravity or immobilization, diminished mechanosignaling exacerbates bone loss, underscoring osteocytes’ role in Wolff’s law—bone adapts to the loads it endures (Ma et al., 2023). Recent studies in single-cell analysis have identified diverse osteocyte subpopulations (Youlten et al., 2021). Notably, some subpopulations display high expression genes linked to neuronal network assembly. This finding broadens current insight into how bone responds to shifts in its local environment.

### 3.2 Regulation of bone remodeling signals

Osteocytes govern remodeling through a sophisticated secretome, balancing formation and resorption (Bolamperti et al., 2022). SOST, a Wnt antagonist, is downregulated by mechanical loading to permit osteoblast activation, while its upregulation in unloading inhibits bone accrual; anti-sclerostin therapies exploit this for anabolic effects (Knowles et al., 2023). RANKL, secreted by osteocytes, drives osteoclastogenesis by binding RANK on precursors, with OPG acting as a decoy receptor to temper this; the RANKL/OPG ratio thus fine-tunes resorption, as seen in osteocyte-specific RANKL deletions attenuating bone loss (Udagawa et al., 2021; Yoshimoto et al., 2022). FGF23, an endocrine regulator, maintains phosphate homeostasis by suppressing renal reabsorption and vitamin D activation, linking skeletal metabolism to systemic mineral balance (Michigami, 2022; Han et al., 2018). These molecules help coordinate the activities of osteoblasts and osteoclasts, making sure that areas of bone resorption are properly restored. Signals released from osteocytes keep this process synchronized by means of paracrine feedback loops. If these regulatory loops are disturbed, either with aging or under disease conditions, bone remodeling can become abnormal (Ru and Wang, 2020) (Figure 1).

### 3.3 Osteocyte interactions with the bone marrow microenvironment

**FIGURE 2:**
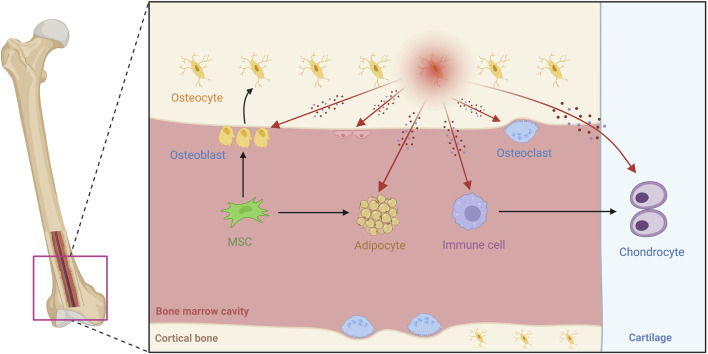
Overview of osteocyte biology and microenvironmental interactions in bone tissue. This schematic diagram shows the bone microenvironment, illustrating how mesenchymal stem cells (MSCs) differentiate into osteoblasts and mature into osteocytes. The image shows the widespread distribution of osteocyte dendritic processes. The image covers a variety of cell types, with osteocytes communicating with osteoblasts, osteoclasts, endothelial cells, bone marrow adipocytes, and immune cells, among others. These interactions occur through paracrine factors such as Sost, RANKL, and FGF23, as well as direct physical contact. Different anatomical regions are labeled, such as cortical bone, articular cartilage, and the bone marrow cavity. The image reveals the diversity of cell populations within the bone and the organization of the specialized microenvironment, with arrows marking the pathways of interaction between osteocytes and other cells.

Osteocytes extend beyond the matrix, engaging in “crosstalk” with immune, and stromal elements in the marrow niche (Elango et al., 2018; Cao H. et al., 2020). Perivascular osteoblasts secrete angiogenic factors, such as VEGF, which affect endothelial cells to maintain nutrient supply or cancer metastasis (Chen et al., 2017; Mulcrone et al., 2020). Through MYD88-dependent signaling in response to PAMPs, these cells are able to attract macrophages and T cells, which can intensify inflammation during infection or in arthritis (Yoshimoto et al., 2022). Their engagement with the surrounding matrix includes perilacunar remodeling; in this process, osteocytes use enzymes like MMP13 to break down the local extracellular matrix, allowing them to adjust to metabolic changes (Mazur et al., 2019). Mitochondrial transfer from osteolineage cells to myeloid populations further regulates immune-mediated bone turnover (Ding et al., 2024). Transfer of osteocyte mitochondria to transcortical vascular endothelial cells accelerates angiogenesis and promotes the repair of cortical bone defects (Liao et al., 2024). Mechanically strained osteocyte-derived exosomes containing miR-3110-5p and miR-3058-3p were transported to osteoblasts, accompanied by increased PGE2, IGF-1 and NOS activities, thereby promoting the osteoblastic differentiation (Zhu Y. et al., 2024). Exosomes secreted by osteocytes under mechanical stress disrupt chondrocyte mitochondrial autophagy through miR-23b-3p, promote cartilage breakdown and inhibit synthesis, thereby accelerating the progression of osteoarthritis (Liu et al., 2025). Downregulation of miR-494-3p in extracellular vesicles derived from senescent osteocytes inhibits osteogenic differentiation and accelerates age-related bone loss *via* the PTEN/PI3K/AKT pathway (Yao et al., 2024). These interactions highlight the role of osteocytes in microenvironmental homeostasis (Figure 2) and have important implications for therapies targeting microenvironmental dysregulation in osteoporosis or metastasis.

## 4 Osteocyte dysfunction in osteoporosis

Osteoporosis is characterized by reduced bone mass and deterioration of bone microarchitecture, resulting from an imbalance in bone remodeling whereby bone resorption exceeds bone formation. Recent studies suggests that osteocyte dysfunction is the important link connecting cell death, signaling abnormalities, metabolic alterations, and systemic factors such as estrogen deficiency and inflammation.

### 4.1 Osteocyte dysfunction in osteoporosis

In osteoporosis, several forms of regulated cell death (RCD) impair osteocyte survival, including apoptosis, ferroptosis, and pyroptosis, each of which perturbs bone homeostasis and promotes bone loss (Ru and Wang, 2020; Zhao et al., 2025). Dying osteocytes release RANKL, which stimulates osteoclast activity (Zhao et al., 2025; Cheng et al., 2022; Sfei et al., 2022). Ferroptosis, an iron-dependent form of cell death driven by lipid peroxidation, occurs more frequently in osteoporosis linked to diabetes or aging. Loss of glutathione peroxidase 4 (GPX4) weakens cellular antioxidant capacity, leaving osteocytes vulnerable to oxidative injury. GPX4 prevents ferroptosis by enzymatically reducing lipid hydroperoxides, while ferrostatin-1 is a pharmacological antioxidant that mimics this protective effect by chemically blocking lipid peroxidation. The protective effect of ferrostatin-1 further supports the contribution of ferroptosis to bone loss (Wu X. et al., 2024; Yang et al., 2022). Pyroptosis, inflammasome-mediated lysis *via* NLRP3 and gasdermin D, is implicated in inflammatory osteoporosis, releasing IL-1β to exacerbate resorption (Zhao et al., 2022). Together, these RCD pathways form a “death-to-resorption” axis, in which apoptotic bodies and DAMPs derived from ferroptotic or pyroptotic cells promote osteoclastogenesis (Zhao et al., 2025; Li C. et al., 2025).

Disruption of the LCN further weakens osteocyte function. Reduced connectivity limits mechanosensation and molecular transport (Tiede-Lewis and Dallas, 2019; Schurman et al., 2021). Aging or glucocorticoid exposure reduces canalicular density and connectivity, impairing fluid shear stress transmission (Yılmaz et al., 2025; Rodriguez et al., 2025). LCN fragmentation is associated with lacunar mineralization and greater fracture susceptibility (Yu et al., 2020). Importantly, alterations in the LCN appear before cortical porosity develops in osteoporotic bone, underscoring the essential role of network integrity in skeletal adaptation (van Tol et al., 2020; Vahidi et al., 2021).

### 4.2 Osteocyte-derived signaling molecules in osteoporosis

Osteocytes regulate bone metabolism through secreted factors, which show marked dysregulation in osteoporosis. Sclerostin, a Wnt/β-catenin inhibitor from mature osteocytes, suppresses osteoblast differentiation and bone formation (Zhang et al., 2023). Estrogen deficiency, as seen in postmenopausal osteoporosis, increases sclerostin expression, suppressing Wnt-driven bone formation and inducing osteocyte apoptosis (Reppe et al., 2015). Epigenetic changes, such as SOST promoter methylation, further reduce osteogenic transcriptional activity in postmenopausal osteoporosis (Shan et al., 2019). FGF23, a phosphatonin, rises in osteoporotic bone, disturbing phosphate balance and impairing mineralization (Dallas et al., 2013; Michigami, 2023). RANKL expression in osteocytes also escalates, driving osteoclast maturation and activity *via* the RANK/RANKL/OPG axis. These shifts are amplified by aging, where senescent osteocytes accumulate, releasing pro-inflammatory cytokines like IL-6 and TNF-α that further upregulate RANKL and sclerostin (Wang et al., 2019; Florencio-Silva et al., 2015). Inflammatory conditions and oxidative stress enhance this dysregulation by inducing apoptosis and releasing DAMPs, which sustain resorption signals (Yu et al., 2017; Yan et al., 2023). Endocrine imbalance, particularly low estrogen, also links directly to ferroptosis and bone fragility (Jiang et al., 2024).

### 4.3 Osteocyte metabolic abnormalities and interactions with bone marrow fat and inflammation

Beyond signaling perturbations, osteocyte metabolic abnormalities in osteoporosis intertwine with bone marrow adipose tissue (BMAT) expansion and an inflammatory microenvironment, fostering a vicious cycle of metabolic reprogramming, oxidative stress, and inflammatory factor release. Osteocytes experience notable changes in their metabolism, often shifting toward glycolysis instead of relying mainly on oxidative phosphorylation. This metabolic adaptation helps these cells survive in low-oxygen environments, but it can also result in increased production of ROS, which in turn impairs mitochondrial function and encourages cellular aging (Srivastava et al., 2022; Bertels et al., 2024). Such reprogramming has been associated with the buildup of BMAT; lipids released from adipocytes can accumulate in the marrow, where they inhibit the formation of new osteoblasts and stimulate osteoclast activity, partly through adipokines such as leptin and adiponectin (Xiao et al., 2024). Oxidative stress further complicates this relationship: higher ROS levels in osteocytes can drive lipid peroxidation and ferroptosis-like cell death, releasing factors that attract macrophages and sustain a bone-resorbing environment (Zhang et al., 2023). In both aging and estrogen deficiency, these metabolic changes in osteocytes have also been linked to systemic consequences like sarcopenia, largely due to interactions between bone and muscle (Choi et al., 2024; He et al., 2020).

Together, these mechanisms place osteocytes at the center of osteoporosis development, where they integrate hormonal, inflammatory, and age-related cues with various metabolic disturbances. Malfunctioning osteocytes not only disturb bone remodeling locally but also exert influence on other organs through endocrine signaling, demonstrating their broader role in physiology. Nonetheless, there are still many uncertainties regarding how these changes unfold over time and across different bone regions, highlighting the need for improved experimental models to clarify osteocyte involvement in osteoporosis progression and guide targeted therapies.

## 5 Osteocyte in bone remodeling and regeneration

Embedded in the mineralized matrix, osteocytes are central to skeletal homeostasis. They integrate mechanical, biochemical, and hormonal cues to guide remodeling and repair, ensuring structural integrity and adaptation. Dysregulated signaling in these cells contributes to disorders such as osteEmbedded in the mineralized matrix, osteocytes are central to skeletal homeostasis.

### 5.1 Role of osteocytes in the bone remodeling cycle

Bone remodeling is a continuous process maintaining skeletal mass and architecture, involving coordinated resorption by osteoclasts and formation by osteoblasts within basic multicellular unit (BMU). Osteocytes initiate and regulate this cycle by sensing microenvironmental changes and mediating coupling between resorption and formation phases (Bolamperti et al., 2022; Niedźwiedzki and Filipowska, 2015). As mechanosensory, osteocytes detect fluid shear stress and matrix deformation *via* their dendritic processes and primary cilia, transducing these into biochemical signals that modulate remodeling (Qin et al., 2020; Choi et al., 2021). In the initiation phase, osteocyte apoptosis—triggered by microdamage, unloading, or glucocorticoid exposure—releases DAMPs and cytokines like IL-6, attracting osteoclast precursors and activating resorption (Bellido, 2014). Osteocytes help maintain the health of their local environment by releasing matrix metalloproteinases (MMPs), which play a part in perilacunar remodeling. Through this process, they keep canaliculi open and support the diffusion of nutrients (Li et al., 2021). Coupling mechanisms ensure formation follows resorption; osteocytes release factors like TGF-β from resorbed matrix, recruiting osteoblast progenitors, while downregulating sclerostin to activate Wnt signaling and promote osteogenesis (Cao W. et al., 2020). When these activities are disrupted—for example, as a result of aging—remodeling becomes uncoordinated, further demonstrating the central role osteocytes play in sustaining the microenvironment. Building on this foundation, osteocytes also direct adaptive responses to external conditions such as mechanical loading or unloading, further shaping skeletal integrity.

### 5.2 Key functions of osteocytes in adaptive bone remodeling

Bone adapts to mechanical demands through Wolff’s law, with osteocytes as primary sensors translating physical stimuli into molecular responses that adjust mass and geometry (Hughes et al., 2020; Wang et al., 2022). Under loading (e.g., exercise), fluid flow activates integrin-αvβ3 and Piezo1 channels, triggering Ca^2+^ influx and downstream pathways like ERK/MAPK, which downregulate sclerostin and upregulate Wnt ligands for enhanced osteogenesis (Wang et al., 2022). Concurrently, loading suppresses RANKL, inhibiting resorption and promoting perilacunar matrix mineralization *via* DMP1 and MEPE (Choi et al., 2021; Klein-Nulend et al., 2012). In contrast, unloading—as in microgravity or bed rest—induces osteocyte senescence and apoptosis, elevating sclerostin and RANKL, leading to uncoupled resorption and bone loss (Hu et al., 2014; Man et al., 2022). Molecularly, reduced mechanotransduction disrupts cytoskeleton-integrin linkages, activating NF-κB and oxidative stress pathways, with upregulated FGF23 exacerbating phosphate waste (Sonawane et al., 2025). Spaceflight studies show that microgravity alters the LCN, impairing fluid and nutrient transport (Man et al., 2022). Osteocytes adapt to environmental change through several mechanisms (Hughes et al., 2020): first, they participate in random (stochastic) remodeling to help maintain bone; second, they are involved in repairing small areas of damage; third, they respond to inactivity by encouraging bone resorption; and finally, they contribute to bone formation in response to mechanical loading. Osteocytes use their cytoskeleton, composed of actin filaments and microtubules, to integrate signals and maintain skeletal health. Looking ahead, new technologies such as organoids and *in vivo* imaging may help clarify how these processes change over time and space, which could eventually support the design of therapies that mimic healthy mechanical signaling.

### 5.3 Osteocytes in fracture healing and bone regeneration: regulation of bone repair, angiogenesis, and stem cell recruitment

Fracture healing takes place in several phases, starting with inflammation, followed by soft callus formation, hard callus ossification, and ultimately remodeling. Osteocytes, both at the injury site and surrounding area, are not passive during these events; instead, they influence each phase by adjusting local inflammation, guiding the entry of new blood vessels, and affecting how progenitor cells behave (Choy et al., 2020). Upon fracture, mechanical disruption induces osteocyte apoptosis, releasing pro-inflammatory signals, which recruit macrophages and initiate hematoma formation (Bahney et al., 2019). Surviving osteocytes upregulate hypoxia-inducible factor-1α (HIF-1α) in response to local hypoxia, promoting VEGF expression to drive angiogenesis essential for nutrient supply and progenitor influx (Bixel et al., 2024; van Brakel et al., 2024). The connection between blood vessel growth and new bone formation is especially important. Osteocytes produce signals such as PDGF-BB and endothelin-1, which promote the growth and maturation of endothelial cells, while type H vessels present in the callus region foster the development of osteoprogenitors (Grosso et al., 2017). In the later stages, as the bone remodels, osteocytes detect how mechanical forces shift within the callus. In response, they alter RANKL and OPG levels, which helps shape the newly formed bone and restore the cortex (Iaquinta et al., 2019). Recently, experimental approaches using exosomes derived from mesenchymal stem cells to target osteocyte pathways have shown promise in boosting bone repair, reflecting the far-reaching, hormone-like roles that osteocytes take on during healing.

## 6 Therapeutic targeting of osteocytes in osteoporosis and bone disease

Through a combination of paracrine signaling, detection of mechanical forces, and control of both osteoclast and osteoblast activity, osteocytes direct the ongoing remodeling of bone. When the regulatory role of osteocytes is disrupted, it leads to osteoporosis and other bone diseases characterized by decreased bone strength and increased risk of fractures. Traditionally, most treatments for osteoporosis have targeted osteoclasts (to reduce bone resorption) or osteoblasts (to enhance bone formation). However, osteocytes stand at the center of this balance, as they coordinate both sides by secreting sclerostin, RANKL, and other mediators. This places osteocytes not as isolated players, but as master regulators of the osteoblast–osteoclast axis. Progress in this field has therefore brought increasing attention to osteocytes as therapeutic targets, with new treatments focusing on blocking osteocyte-derived molecules, exploring tissue regeneration, and moving toward precision medicine. The following sections highlight representative clinical and preclinical advances.

### 6.1 Sclerostin inhibitors and novel bone anabolic agents

Sclerostin, secreted by osteocytes, inhibits Wnt/β-catenin signaling and suppresses bone formation. Neutralizing sclerostin has emerged as a powerful anabolic strategy for osteoporosis (Ke et al., 2012). Romosozumab, a monoclonal antibody targeting sclerostin, promotes bone accrual by enhancing osteoblast activity while transiently reducing resorption. Phase 3 trials, including FRAME and ARCH, demonstrated significant increases in bone mineral density (BMD) at the spine and hip with fracture risk reductions for vertebral fractures over 12 months (Lewiecki, 2020). Recent real-world studies from 2024 to 2025 further confirmed its efficacy in postmenopausal women, reporting BMD increases of 6.58%–14.65% at lumbar spine and femoral neck after 12 months, especially when sequenced after denosumab to prevent rebound bone loss (Park et al., 2025; Piasentier et al., 2025). Moreover, microarchitectural improvements, assessed *via* high-resolution peripheral quantitative CT, reveal enhanced trabecular connectivity and cortical thickness (McClung et al., 2025). Beyond romosozumab, bispecific antibodies combining sclerostin inhibition with RANKL blockade are under development, aiming for dual anabolic–antiresorptive effects in preclinical studies (Xu et al., 2023; Elahmer et al., 2024). It is worth noting that other clinical-stage therapies, including the PTH analogs teriparatide and abaloparatide, as well as the anti-RANKL antibody denosumab, have already shown proven efficacy in reducing fracture risk and improving bone mass. These agents primarily act by stimulating osteoblast activity (teriparatide/abaloparatide) or suppressing osteoclast function (denosumab) (Ebina et al., 2025; Bone et al., 2017). In this therapeutic landscape, sclerostin inhibitors like romosozumab are distinctive in directly targeting osteocyte-derived signals, thereby complementing existing approaches and broadening options for individualized osteoporosis management.

### 6.2 Stem cell and gene editing therapies: prospects for osteocyte-targeted regenerative medicine

Regenerative strategies focus on rebuilding osteocyte networks and restoring bone balance, with approaches that use stem cells as well as gene-editing technologies. MSCs, sourced from either bone marrow or fat tissue, can differentiate into osteocytes and also release growth factors such as BMPs, which contribute to bone repair (Dalle Carbonare et al., 2025; Chu et al., 2024). In some models of osteoporosis, MSCs engineered to produce more PDGFB have been shown to boost the formation of trabecular bone and increase bone strength, with reports of up to 45% greater bone volume (Chen et al., 2015). Researchers have also used CRISPR-Cas9 gene editing to specifically alter genes expressed in osteocytes, including those involved in sclerostin regulation and mechanosensing pathways (He et al., 2017; Michalski and Williams, 2023). Some preclinical work has transplanted iPSCs modified by CRISPR into bone defects, resulting in the generation of mature, functional osteocytes that enhance both mineralization and blood vessel growth within the repaired tissue (Iaquinta et al., 2019). Delivering edited MSCs *via* exosomes has also been explored as a way to strengthen paracrine signaling, lower inflammation, and support new bone formation in osteoporosis models (Chen Y. et al., 2024). One study showed that mesenchymal stem cells combined with strontium-containing scaffolds enhanced cell attachment and promoted bone growth in osteoporotic rats (Wu et al., 2020). In a key study, AAV gene therapy targeting SHN3 in bone reversed bone loss in osteoporosis models (Lin et al., 2024). Genetically modified stem cell therapy is a safe and effective method that can significantly improve BMD and BV/TV in animal models of osteoporosis (Huang et al., 2025). While these approaches are promising, they face obstacles such as immune rejection of transplanted cells, off-target gene editing effects, and scalability for human applications.

### 6.3 Multi-omics, AI, and personalized precision therapy

Multi-omics integration—encompassing genomics, proteomics, and metabolomics—unveils osteocyte-driven biomarkers for osteoporosis. Genome-wide association studies identify variants in osteocyte genes like SOST, influencing BMD and fracture susceptibility (Li et al., 2024). Proteomic studies have identified higher levels of sclerostin and DKK1 as markers that may help predict bone health, while analyses of cellular metabolites have pointed to abnormal lipid patterns in aging osteocytes (Wang et al., 2024; Yuan et al., 2024). Recently, machine learning and other AI-based tools have begun to combine these biological datasets for risk prediction, often providing a more precise estimate of fracture risk than what is possible with standard DXA imaging (Saleem and January 2024; Mis et al., 2025). Deep learning on multi-modal datasets stratifies patients for personalized therapies.

## 7 Perspectives and future directions

Recent studies highlight how osteocytes employ diverse molecular sensors—including the cytoskeleton, primary cilia, integrins, and ion channels such as Piezo1—to convert mechanical forces into biochemical signals that regulate remodeling and structural adaptation. This complexity explains their central role not only in osteoporosis but also in conditions such as osteoarthritis and bone metastasis, where impaired mechanotransduction aggravates disease. Currently, a growing number of studies are employing systems biology approaches to elucidate the functions of osteoblasts. Single-cell RNA sequencing, spatial transcriptomics, and artificial intelligence technologies are helping us to more precisely map osteoblast heterogeneity and the LCN (Tong et al., 2024). Improvements in live imaging and organ-on-a-chip platforms also provide opportunities to study osteocyte–osteoblast–osteoclast interactions under mechanical load. There is increasing recognition of osteocyte-endocrine effects on muscle and kidney—which could open new perspectives on aging and whole-body disease. However, major gaps remain in human *in vivo* research, as most data are derived from animal and preclinical models. High-resolution imaging, single-cell, and spatial omics are essential for characterizing osteocyte biology in the human skeleton. Moving forward, teamwork across disciplines, especially between bioengineering and pharmacology, will be essential for turning basic discoveries about osteocytes into tailored therapies that boost regeneration and bone health.
